# Toxicokinetics of a Single Oral Dose of Aflatoxin B_1_ in Plasma, Feces, and Urine of Male Donkeys

**DOI:** 10.3390/toxins17040206

**Published:** 2025-04-20

**Authors:** Yulong Feng, Min Li, Yunduo Zheng, Honglei Qu, Pengshuai Li, Boying Dong, Yantao Wang, Guangyuan Liu, Bin Jia, Qiugang Ma

**Affiliations:** 1 College of Animal Science and Technology, Shihezi University, Shihezi 832003, China; fengyulong19871024@163.com; 2 Shandong Key Laboratory of Gelatine Medicines Research and Development, Dong’e Ejiao Co., Ltd., Liaocheng 252201, China; limin3@dongeejiao.com (M.L.);; 3 State Key Laboratory of Animal Nutrition and Feeding, College of Animal Science and Technology, China Agricultural University, Beijing 100193, China

**Keywords:** toxicokinetics, mycotoxins, aflatoxin B_1_, male donkeys

## Abstract

Aflatoxin B_1_ (AFB_1_) is widely present in raw materials for food and feedstock, posing a significant threat to the health of humans and animals. This study explored the toxicokinetics of a single oral administration of AFB_1_ at a dose of 100 µg·kg^−1^ BW (body weight). Donkey blood samples were gathered at 0, 5, 10, 15, 20, 30, 45, and 60 min and at 1.5 h, 2 h, 2.5 h, 3 h, 3.5 h, 4 h, 4.5 h, 6 h, 9 h, 12 h, 24 h, 48 h, 72 h, 96 h, and 120 h through jugular vein sampling needles at intervals. Fecal and urinary samples were collected at 0 h and every 6 h thereafter until 120 h. The concentrations of AFB_1_ and AFM_1_ in plasma, urine, and feces were quantitatively analyzed using LC-MS/MS. The maximum concentrations of AFB_1_ and AFM_1_ in plasma were 13.10 ± 6.35 µg·L^−1^ and 0.72 ± 0.33 µg·L^−1^, occurring at 1.38 ± 0.89 h and 2.25 ± 1.57 h after oral administration, respectively. The AFB_1_ and AFM_1_ elimination half-lives (T_1/2_Elim) were 6.65 ± 2.84 h and 5.85 ± 3.00 h, respectively. The total clearances (CL) of AFB_1_ and AFM_1_ were 163 ± 52.2 L·kg^−1^ BW^−1^·h^−1^ and 3210 ± 2450 L·kg^−1^ BW^−1^·h^−1^, and the volumes of distribution (Vd) for AFB_1_ were 1440 ± 417 L·kg^−1^·BW and 22,400 ± 14,800 L·kg^−1^·BW, respectively. In addition, the total amounts of AFB_1_ and AFM_1_ excreted over 120 h through urine and feces accounted for 3.38 ± 0.92% and 3.44 ± 1.45% of the total intake, respectively (calculated by material mass). Furthermore, the research showed that the absorption and metabolism of AFB_1_ were rapid in male donkeys, with the tissue exhibiting a wide distribution and long duration.

## 1. Introduction

Aflatoxin B_1_ (AFB_1_) was first discovered in the 1960s and caused the deaths of hundreds of thousands of turkeys in the UK [1,2]. AFB_1_ is produced by *Aspergillus flavus* and *Aspergillus parasiticus* during growth, storage, and processing and is widely present in corn, wheat, sorghum, rice, oats, rye, barley, soya, peanuts, and milk [3,4,5]. AFB_1_ poses a real threat to animal and human health [6]. Generally, the main route of AFB_1_ exposure is dietary, with minimal dermal and inhalation exposure [7]. AFB_1_ is transported to the liver through blood circulation for metabolism [8]. When the P450 enzyme system metabolizes AFB_1_, it produces the final carcinogen, AFB_1_-8,9-epoxide (AFBO). AFBO covalently bonds to proteins and DNA to form adducts and causes DNA mutations that can lead to tumorigenesis [9].

After entering the body orally, AFB_1_ is rapidly absorbed through the intestine. It then enters the bloodstream through hepatic–intestinal circulation and is metabolized and excreted as AFB_1_ and AFM_1_ through urine. Another portion is metabolized through the intestine and microbiota and excreted as AFB_1_ and AFM_1_ through feces.

Many animal diseases are caused by the consequences of AFB_1_ exposure, and it can affect vaccine-induced immunity [10,11]. Studies have reported that AFB_1_ causes hazardous toxicity and leads to several changes in plasma biochemical parameters [12]. Furthermore, the toxicokinetics of AFB_1_ have been studied in different kinds of animals, including ewes [13], rats [14], cows [3], monkeys [15], and equines [16]. Among different species, the toxicokinetic parameters of AFB_1_, including the peak time, half-life, and clearance rate, are significantly diverse. Previous research has shown that equines are sensitive to AFB_1_, with clinical signs and biochemical changes observed at various doses [16]. AFB_1_ can be metabolized into aflatoxin M_1_ (AFM_1_), AFB_1_-DNA, AFB_1_-lysine, and other metabolites. AFM_1_ is commonly present in cattle, milk, and humans, leading to many potential dietary exposure pathways [17]. According to reports, the incidence and contamination levels of AFM_1_ in milk samples were high [18,19,20]. Since 2002, the International Agency for Research on Cancer (IARC) has upregulated AFM_1_ from a Class 2B carcinogen to a Class 1 carcinogen [21]. AFB_1_-DNA [22] and AFB_1_-lysine are often used as biomarkers for AFB_1_ exposure [23,24]. AFB_1_-DNA adducts are considered the only intermediate product involved in AFB_1_-induced liver cancer [25]. The AFB_1_-lysine adduct in the blood is believed to reflect prolonged exposure, based on the longer half-life of human albumin compared to urinary metabolites, which reflects short-term excretion. Therefore, simultaneous exposure to these toxins poses a significant hazard [26].

Donkey meat and dairy products are increasingly favored by people in China due to their high nutritional value [27,28,29,30,31]. In addition, to our knowledge, there have been studies on the toxicokinetics of vomitoxins [32], ochratoxin [33], and zearalenone in donkeys [34]. Compared to other species, there are few reports on donkeys in terms of of AFB_1_. This study investigates the toxicokinetics of AFB_1_ after administering a single oral dose of aflatoxin B_1_ in male donkeys to estimate the metabolic degradation time of AFB_1_ in donkeys. Based on the results, suggestions can be provided to ensure food security.

## 2. Results

### 2.1. Detection Method Validation

**Figure 1:**
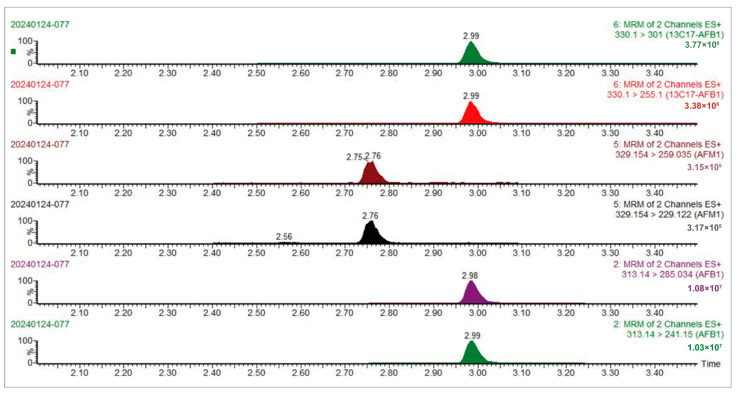
The MRM chromatogram of the plasma samples for AFB_1_, AFM_1_, and ^13^C_17_-AFB_1_.

A six-point, 1/x-weighted calibration curve (ACE/water, 50:50, *v*/*v*) was prepared from the mixture of target analytes (0.1~200 ng·mL^−1^) with 5 ng·mL^−1^ of ^13^C_17_-AFB_1_, and the determination coefficient (R^2^) for each analyte was greater than 0.99. The isotope internal standard dilution method was utilized for quantification, and sensitivity, accuracy, and precision tests were performed on each sample matrix. The limit of quantification (LOQ, 10 × signal-to-noise) for AFB_1_ and AFM_1_ was 0.5 ng·mL^−1^ for urine, 0.1 ng·mL^−1^ for plasma, and 1.0 ng·g^−1^ for fecal samples, respectively. The recovery rates of standard samples in various matrices were higher than 80.8%, with the relative standard deviation (RSD) lower than 13.6%. The detailed verification results are shown in Table 1 and Figure 1. Additionally, a chromatogram for the analytes in a plasma sample is presented in Figure 1.

The recovery and relative standard deviation (RSD) in plasma, feces, and urine were validated at low, medium, and high concentrations of AFB_1_.

### 2.2. Plasma Toxicokinetic Parameters of AFB_1_ and AFM_1_

None of the donkeys employed showed noticeable adverse effects on their health during the experimental period. As shown in Table 2, the average weight of the male donkeys was 124.25 ± 2.75 kg. After a single oral administration of AFB_1_ (100 µg·kg^−1^·BW), AFB_1_ entered the circulatory system and was absorbed.

Plasma samples were collected regularly to detect the concentrations of AFB_1_ and AFM_1_. Non-compartmental methods were used to evaluate the toxicokinetic parameters of AFB_1_. The concentration of AFB_1_ in plasma reached its peak concentration (Cmax = 15.6 ± 6.88 µg·L^−1^) at 1.38 ± 0.89 h (Tmax) after administration. The elimination half-life (T_1/2_Elim) was 6.65 ± 2.84 h. The area under the plasma concentration–time curve (AUC_0-inf_) was 82.6 ± 24.3 µg·L^−1^·h. The total plasma clearance rate was 163 ± 52.2 L·kg·BW^−1^·h^−1^. In addition, the volume of distribution was 1440 ± 417 L·kg^−1^·BW.

The AFM_1_ concentration in plasma reached a peak concentration (Cmax = 0.72 ± 0.33 µg·L^−1^) at 2.25 ± 1.57 h after oral administration (Table 2). The elimination half-life of AFM_1_ was calculated as 5.85 ± 3.00 h, the area under the plasma concentration–time curve (AUC_0-inf_) was 5.50 ± 2.93 µg·L^−1^·h, and the total plasma clearance (Cl) was 3210 ± 2450 L·kg·BW^−1^·h^−1^. The volume of distribution (Vd) was 22,400 ± 14,800 L·kg^−1^·BW.

### 2.3. Plasma Concentration of AFB_1_ and AFM_1_

**Figure 2:**
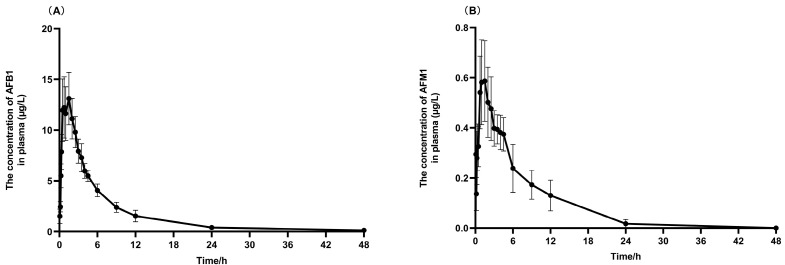
The average concentration time distribution of AFB_1_ and AFM_1_ in the plasma. This graph shows the concentration–time curves of AFB_1_ (**A**) and AFM_1_ (**B**) in plasma over time after orally taking 100 µg·kg^−1^·BW; n = 6.

AFB_1_ and AFM_1_ were detected in plasma samples just 5 min after a single dose of gavage administration of AFB_1_ (100 µg·kg^−1^·BW). As shown in Figure 2A, the concentration of AFB_1_ increased, leading to a peak concentration (Cmax) of 15.6 ± 6.88 µg·L^−1^, while the time to reach the peak concentration (Tmax) was 1.38 ± 0.89 h. Meanwhile, as depicted in Figure 2B, the concentration of AFM_1_ in plasma was lower compared to that of AFB_1_, with a maximum concentration (Cmax) of 0.72 ± 0.33 µg·L^−1^, and it reached the peak (Tmax) at 2.25 ± 1.57 h.

### 2.4. Recovery of AFB_1_ and AFM_1_ in Urine and Feces

**Figure 3:**
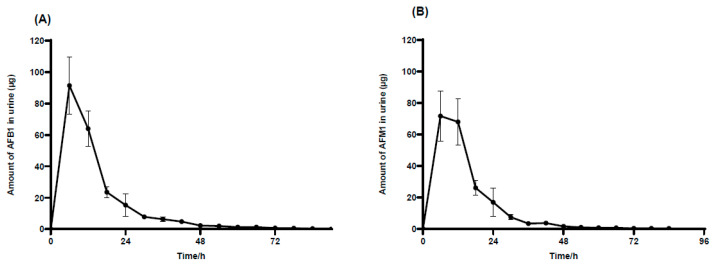
The average excretion of AFB_1_ and AFM_1_ in the urine of male donkeys. This graph explains the average excretion of unbound AFB_1_ (**A**) and AFM_1_ (**B**) in urine with time in male donkeys orally taking AFB_1_ at 100 µg·kg^−1^·BW, n = 6.

As depicted in Figure 3A, AFB_1_ was detected 6 h after oral administration, and the excretion subsequently peaked in urine. Thereafter, AFB_1_ excretion significantly decreased from 6 to 24 h, and only trace levels of AFB_1_ were detected after 48 h. Ultimately, AFB_1_ was not detected after 90 h. Figure 3B illustrates that the metabolite AFM_1_ reached its maximum excretion at 6 h post-administration. The excretion of AFM_1_ then slightly decreased from 6 to 12 h, followed by a significant drop from 12 to 30 h. Finally, AFM_1_ was no longer detectable by 84 h.

**Figure 4:**
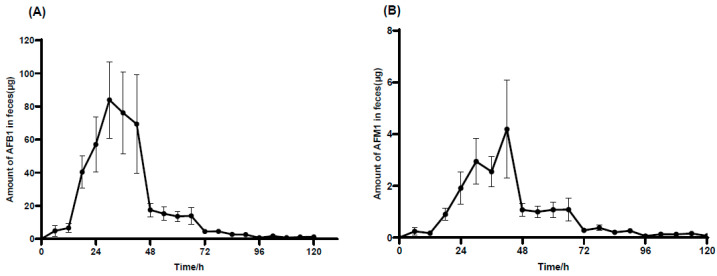
Average AFB_1_ and AFM_1_ excretion in feces of male donkeys. This diagram interprets the mean egestion of unconjugated AFB_1_ (**A**) and AFM_1_ (**B**) in feces with time in male donkeys orally taking AFB_1_ at 100 µg·kg^−1^·BW, n = 6.

Figure 4A shows that the excretion of AFB_1_ in feces was detected at 6 h and peaked at 30 h. Subsequently, the excretion declined slowly from 30 to 42 h, followed by a sharp decrease in AFB_1_ excretion after 42 h. The excretion of AFB_1_ was detected in trace levels at 120 h. As shown in Figure 4B, AFM_1_ was detected at 6 h and reached its peak at 42 h in feces. It then began to decrease, experiencing a rapid decline from 42 to 72 h. By 120 h, only tiny quantities of AFM_1_ were detected in feces.

According to Table 3, the single dose of AFB_1_ that was given to the donkeys amounted to 12,420 ± 270 µg. Subsequently, AFB_1_ and AFM_1_ were excreted through both urine and feces. The results indicated that the fecal excretion rate of AFB_1_ and AFM_1_ constituted 3.44 ± 1.45% of the total AFB_1_ intake, whereas the absorption rate was 96.56 ± 1.45%. In contrast, the total amount of AFB_1_ and AFM_1_ that was excreted in the urine accounted for 3.28 ± 0.92% of the total intake.

**Figure 5:**
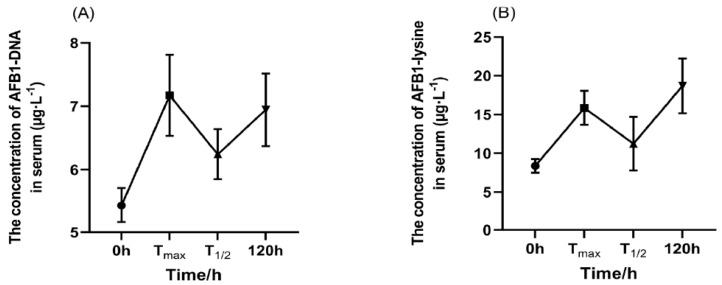
The average concentration of AFB_1_-DNA (**A**) and AFB_1_-lys (**B**) changed in the serum of male donkeys after oral administration of 100 µg·kg^−1^·BW of AFB_1_, n = 6.

The concentrations of AFB_1_-DNA and AFB_1_-lysine in blood were also found with some fluctuation. According to Figure 5A,B, these concentrations reached their first peak at the test Tmax and subsequently decreased. At T_1/2_Elim, the concentration of AFB_1_-DNA and AFB_1_-lysine in blood was the lowest at 6.24 µg·L^−1^ and 11.24 µg·L^−1^, respectively. The concentrations once again reached 6.95 µg·L^−1^ and 18.69 µg·L^−1^ at 120 h.

## 3. Discussion

Historically, donkeys were a source of agricultural labor and pet companions and provided high-quality meat, dairy products, and raw materials for traditional Chinese medicine [35,36]. Previous studies have shown that the toxicokinetics of AFB_1_ significantly vary among animals, including the process of absorption, clearance, and excretion. At present, there has been no research on the toxicokinetics of AFB_1_ ingestion in donkeys, whether through oral or other administration methods. This study utilized donkeys as subjects to investigate the toxicokinetics of AFB_1_ and aimed to clarify its pharmacokinetics following ingestion. As indicated in Table 4, the donkeys exhibited more extensive and pronounced toxicokinetic parameters compared to other animals.

After being orally administered a 100 µg·kg^−1^·BW AFB_1_ dose, it was quickly absorbed into the body and reached its peak concentration rapidly. The peak concentration of AFB_1_ in this study was 15.6 ± 6.88 µg·L^−1^ (Cmax), and the peak time occurred at 1.38 ± 0.89 h (Tmax). Compared to humans (Tmax = 1.02 ± 0.31 h) [37], broilers (Tmax = 0.3 ± 0.1 h) [38], rats (Tmax = 0.17 h) [14], and cows (Tmax = 0.42 h, Tmax = 0.58 ± 0.17 h) [3], the peak concentration for donkeys (Tmax = 1.38 ± 0.89 h) was longer and reflected a slower rate of absorption. The AFB_1_ levels in plasma were different and might have been caused by highly individual variability, probably related to differences in plasma volume or the other passage of toxins through the gastrointestinal mucosa [39]. In addition, studies on cows have shown a terminal elimination half-life (T_1/2_) of 15.5 ± 0.51 h and 1.28 h in pregnant mice [40]. These findings indicated a longer T_1/2_ (6.65 ± 2.84 h) in donkeys compared to pregnant mice but a significantly shorter one than in cows. Previous studies have shown that cows, beef, and cattle were more susceptible to AFB_1_ than sheep or equines, which was consistent with the results of this study [41].

Many published papers have shown that when cows were fed with 4 μg·kg^−1^·BW of AFB_1_, AFM_1_ reached its peak concentration (0.5 ± 0.1 µg·L^−1^) at 4 h [42]. In contrast, in this study, the AFM_1_ peak time for male donkey plasma was 2.25 ± 1.57 h (0.72 ± 0.33 µg·L^−1^). Compared to cows, donkeys exhibited a longer Tmax for AFM_1_ concentration in the plasma. Additionally, the plasma ratio of AFM_1_ to AFB_1_ increased from 0 at 0.08 h to 0.07 at 0.16 h and reached 0.09 at 9 h. When the plasma AFB_1_ concentration was below 2.4 µg·L^−1^, AFM_1_ was not detected in the plasma. The phenomenon resulted from the ffact that the conversion of AFB_1_ to AFM_1_ was lower than the body’s ability to clear AFM_1_ through feces and urine, preventing metabolite accumulation in the bloodstream [8]. Furthermore, the donkeys had a lower rate of AFB_1_ conversion to AFM_1_ [3]. The differences in plasma Cmax can be attributed to factors such as species, AFB_1_ intake, gastrointestinal absorption, and health status, particularly the activity of cytochrome P450 (CYP450) enzymes, which play an important role in the conversion of AFB_1_ to AFM_1_ in the liver [43,44].

This study also conducted relevant research on the biomarkers AFB_1_-DNA and AFB_1_-lys adducts in serum [45]. According to previous studies, AFB_1_-DNA was produced by the P450 enzyme system and was metabolized into AFB_1_-8,9-epoxide, which covalently bound to DNA and lysines to form AFB_1_-DNA and AFB_1_-lys, respectively [46]. Furthermore, AFB_1_-DNA adducts were considered the only intermediate products involved in the development of liver cancer [25], while AFB_1_-lys served as a valuable biomarker for long-term exposure [47,48]. In this experiment, AFB_1_-DNA adducts were detected at 0 h, Tmax, half-life, and 120 h. The results showed that the first peak appeared at Tmax and that the half-life was the lowest, but the concentration remained high at 120 h. Additionally, the growing-stage male donkeys exhibited a higher absorption rate of AFB_1_. In this study, the total amount of AFB_1_ excreted in feces and urine was 833.25 μg, and most of the AFB_1_ entered the body and was transported to various parts of the body through body fluids. This research demonstrated that it was bound with serum proteins to form various adducts that remained between tissues and were slowly metabolized.

AFB_1_ is absorbed into the blood through the gastrointestinal tract and then transported by the bloodstream to various tissues. The existing literature has shown that the apparent distribution volume (Vd) of broiler chickens was 18.4 ± 7.7 L·kg^−1^ [38]. Researchers explored the Vd values for toxicokinetics in OTA (0.15 ± 0.036 L·kg^−1^·BW) [33], DON (2.82 ± 0.35 L^−1^·kg·BW) [32], and ZEN (216.17 ± 58.71 L^−1^·kg·BW) on donkeys [34]. In this study, the Vd value was determined to be 1440 ± 417 L·kg^−1^·BW when compared to OTA, ZEN, and DON, suggesting a significantly wider tissue distribution of AFB_1_ in the body. However, this finding needs to be supported by further validation.

Previous studies have shown that AFB_1_ was absorbed through the gastrointestinal tract, was mainly metabolized in the liver, and was excreted through the urine [13]. Moreover, undigested substances in the gastrointestinal tract were excreted through the feces [49]. In this research, the outputs of AFB_1_ and AFM_1_ in the feces and urine were 409.30 ± 172.57 μg and 17.89 ± 8.40 μg, while the figures for lambs and ewes were 210.86 ± 46.39 μg and 195.20 ± 66.62 μg, respectively [50,51]. However, the AFB_1_ excretion rate in male donkeys (6.72%) was higher than in ewes (5.3%). This result may be related to the type and size of the animal, the method of digestion, and the dosage of the toxin.

## 4. Conclusions

In summary, male donkeys demonstrated a high absorption rate of 93.28% following a single oral dose of 100 µg·kg^−1^ BW of AFB_1_, with plasma levels peaking at 1.38 ± 0.89 h and maximum fecal and urinary excretion at 6 h. By 120 h post-administration, the cumulative excretion rates of AFB_1_ and AFM_1_ were 3.44% and 3.28%, respectively. The complex elimination process indicated that AFB_1_ was primarily metabolized in donkeys by rapidly forming other metabolites in the liver, with only a small fraction excreted as AFB_1_ and AFM_1_ in feces and urine.

## 5. Materials and Methods

### 5.1. Chemicals, Products, and Reagents

The standard samples of aflatoxin B_1_ (AFB_1_) and aflatoxin M_1_ (AFM_1_) were acquired from Fermentek (Jerusalem, Israel) for analysis. The ^13^C_17_-aflatoxin B_1_ (^13^C_17_-AFB_1_) isotope-labeled internal standard (IS) was purchased from Romer Labs (Tulln, NÖ, Austria). Additionally, the above standard chemicals were appropriately diluted with acetonitrile (ACN) to prepare 5 µg·mL^−1^ stock solutions, which were then stored in brown glass bottles at temperatures below −18 °C. LC-MS grade ACN, methanol (MeOH), and formic acid (FA) were bought from Thermo Fisher Scientific (Pittsburgh, PA, USA). Moreover, ultrapure water (18.2 MΩ cm) was self-made using the Bedford Millipore Milli-Q system (Bedford, OH, USA). Analytical-grade anhydrous magnesium sulfate (MgSO_4_) and sodium chloride (NaCl) were obtained from Sinopharm Chemical Reagent Co., Ltd. (Beijing, China). Octadecyl-bonded silica (C_18_, 50 μm) and primary–secondary amine (PSA, 40–60 μm) were also acquired from Bonna-Agela Technologies (Tianjin, China). Furthermore, the ELISA quantitative detection kits for AFB_1_-DNA and AFB_1_-lysine adducts were purchased from Hengyuan Biological (Shanghai, China).

### 5.2. Animal and Treatment

This study was conducted according to the Guidelines for Experimental Animals of the Ministry of Science and Technology (Beijing, China) and was approved by the Bioethics Committee of Shihezi University (NO.A 2023-201, 6 March 2023). Six healthy 9-month-old male donkeys, weighing 124.25 ± 2.75 kg, were chosen and were fed in metabolic cages individually to collect fecal and urine samples accurately. Additionally, the metabolic cages provided suitable movement space for the donkeys. After a 5-day acclimation period, overnight fasting was performed, and blood, feces, and urine samples were collected 4 h before the test. AFB_1_ standards were dissolved in DMSO (stock 1 mg·mL^−1^) and were then diluted with normal saline to a working solution at a concentration of 0.5 mg·mL^−1^ [40]. This working concentration was administered as a single dose of 100 µg·kg^−1^·BW and was infused into the donkeys’ pharynx through an oral catheter [52].

### 5.3. Sample Collection

Each time, 10 mL blood samples were gathered from the jugular vein of the donkeys using heparin sodium vacuum tubes before administration (0 min) and then at 5, 10, 15, 20, 30, 45, and 60 min and at 1.5 h, 2 h, 2.5 h, 3 h, 3.5 h, 4 h, 4.5 h, 6 h, 9 h, 12 h, 24 h, 48 h, 72 h, 96 h, and 120 h after oral administration. The blood was then centrifuged at 4500 rpm for 10 min, after which the plasma was separated into 1 mL EP tubes using a pipetting gun. These plasma samples were labeled and then placed in the refrigerator at −20 °C. Additionally, urine volumes and feces weights were collected individually before administration (0 h) and at 6 h, 12 h, 18 h, 24 h, 30 h, 36 h, 42 h, 48 h, 54 h, 60 h, 66 h, 72 h, 78 h, 84 h, 90 h, 96 h, 102 h, 108 h, 114 h, and 120 h after the gavage of the AFB_1_ working solution. Urine samples were then loaded into 15 mL gauge centrifuge tubes, and the feces were mixed and collected into ziplock bags. These samples were then stored at −20 °C. All samples were carefully marked and recorded for further detection and analysis.

### 5.4. Sample Treatment

Mycotoxin extraction and cleanup procedures were conducted as previously reported with a few modifications [53]. Initially, a 500 μL plasma sample was spiked with 10 μL of IS working solution (100 ng·mL^−1^ in ACN) and 1.5 mL of 0.5% FA-ACN (*v*/*v*), followed by vortex mixing for 30 s. Subsequently, 0.2 g of MgSO_4_ and 0.2 g of NaCl were added, and the mixture was shaken for 2 min. The test samples were then centrifuged at 8000 rpm for 5 min. After centrifugation, the entire liquid supernatant was desiccated under a mild nitrogen flow at 50 °C, and the residue was subsequently dissolved in 200 μL of ACN/water (50:50, *v*/*v*).

Next, a 200 μL urine sample was treated with 40 μL of IS working solution (100 ng·mL^−1^ in ACN), followed by the addition of 560 μL of 0.5% FA-ACN (*v*/*v*). This mixture was mixed using a vortex for 30 s and centrifuged at 10,000 rpm for 2 min.

The freeze-dried fecal sample was ground into a fine powder, and then 200 mg was weighed into a 10 mL plastic centrifuge tube. This sample was spiked with 4 μL of IS stock solution (5 μg·mL^−1^ in ACN) and sonicated with 2 mL of 0.5% FA-ACN (*v*/*v*) for 20 min. After sonication, the mixture was centrifuged at 8000 rpm for 5 min at room temperature, yielding a supernatant of 0.5 mL.

Finally, the levels of AFB_1_-DNA and AFB_1_-lys adducts in serum were determined using competitive ELISA kits. The absorbance (OD) value was read at a wavelength of 450 nm, and the adduct content was calculated according to the standard curve.

### 5.5. Standard Solutions

The AFB_1_ and AFM_1_ mixed standard solution working liquid was prepared as follows: with 50% acetonitrile water (ACE/water, 50:50, *v*/*v*) as the solvent, an appropriate amount of AFB_1_ and AFM_1_ single standard solution (100 μg·mL^−1^) was accurately removed and prepared into a AFB_1_ and AFM_1_ mixed standard solution working liquid with a mass concentration of 1 μg·mL^−1^.

The ^13^C_17_-AFB_1_ standard solution working liquid was prepared as follows: with 50% acetonitrile water as the solvent, an appropriate amount of ^13^C_17_-AFB_1_ single standard solution was accurately removed and prepared into a ^13^C_17_-AFB_1_ standard solution working liquid with a mass concentration of 0.1 μg/mL.

The AFB_1_ and AFM_1_ series calibration standard solutions were prepared as follows: With AFB_1_ and AFM_1_ as the targets, ^13^C_17_-AFB_1_ as the internal quantitative standard, 50% acetonitrile water as the solvent, an AFB_1_ and AFM_1_ mixed standard solution working solution, and a ^13^C_17_-AFB_1_ standard solution working solution, the solvent dilution method was adopted. AFB_1_ and AFM_1_ series calibration standard solutions with target concentrations of 0.1, 1, 5, 10, 50, and 200 ng·mL^−1^ and an internal isotope standard fixed at 5 ng·mL^−1^ were prepared.

### 5.6. Method Validation

The quantitative analytical methods were validated according to the validation protocol of Wang et al. The analytes in the samples were qualitatively identified by comparing the abundance ratio of the qualitative ion pairs (±20%) and the retention time (±0.1) with those in standard solutions. ^13^C_17_-AFB_1_ was used as the IS for the quantitation analysis of all target compounds. The concentration corresponding to 3 times the signal-to-noise ratio (S/N) was used as the instrument detection limit (LOD), and the concentration corresponding to 10 times the signal-to-noise ratio (S/N) was used as the instrument quantitative limit (LOQ). Three different levels of mixed toxin standards, i.e., low concentration (2 µg·L^−1^ or 2 µg·kg^−1^), medium concentration (10 µg·L^−1^ or 10 µg·kg^−1^), and high concentration (100 µg·L^−1^ or 100 µg·kg^−1^), were added to blank plasma, urine and fecal samples were used for recovery validation, according to the 5.4 sample pretreatment method, with 5 parallel samples for each concentration, and the recovery rate and precision were calculated.

### 5.7. UPLC-MS/MS Analysis

AFB_1_ and its metabolite AFM_1_ were identified and quantified using a Waters ACQUITY UPLC system coupled with a XEVO TQ-S electrospray triple-quadrupole mass spectrometer (LC-MS/MS) (Milford, CT, USA) operating with negative ESI and in the multiple reactions monitoring (MRM) mode. Detailed instrumental parameters are provided in Table 5.

### 5.8. Statistical Analysis

Statistical analysis was conducted using the standardized concentration of AFB_1_. Subsequently, the toxicokinetic parameters of AFB_1_ in the plasma were calculated using the non-compartmental model of the WinNonlin 5.2.1 software (Certara, Inc., Princeton, NJ, USA). Next, concentration–time curves were drawn based on the mean concentrations of AFB_1_ and its metabolite AFM_1_ in the plasma of the experimental donkeys at different times. Similarly, corresponding excrement curves were also drawn based on the total excretion of AFB_1_ and its metabolite AFM_1_ in urine and feces at various times using GraphPad Prism 9.4.1 (GraphPad Software, Inc., San Diego, CA, USA). Finally, the mean value of the data was represented by ± SEM (the standard error of the mean).

## Figures and Tables

**Table 1:** Recoveries and precisions (n = 5) of the method.

Matrix	Spiked Concentration (µg·L^−1^)/(µg·kg^−1^)	Items (%)	AFB_1_ (%)	AFM_1_ (%)
Urine	2	Rrecovery	85.6	80.8
		RSD	10.3	7.6
	10	Rrecovery	92.7	81.8
		RSD	9.9	5.5
	100	Rrecovery	101.7	100.9
		RSD	6.9	3.7
Feces	2	Rrecovery	88.5	90.8
		RSD	11.4	13.6
	10	Rrecovery	90.1	93.7
		RSD	9.6	8.2
	100	Rrecovery	86.6	83.3
		RSD	11.4	6.4
Plasma	1	Rrecovery	81.7	96.2
		RSD	9.6	10.3
	10	Rrecovery	88.7	83.7
		RSD	5.4	7.2
	50	Rrecovery	92.6	97.5
		RSD	4.8	7.6

**Table 2:** Plasma toxicokinetic parameters of donkeys after oral administration of single-dose AFB_1_.

Toxicokinetic Parameters	Value of AFB_1_	Value of AFM_1_
Body weight (kg)	124.25 ± 2.75	
AFB_1_ (µg·kg^−1^·BW)	100	
Cmax (µg·L^−1^)	15.6 ± 6.88	0.72 ± 0.33
Tmax (h)	1.38 ± 0.89	2.25 ± 1.57
T_1/2_Elim (h)	6.65 ± 2.84	5.85 ± 3.00
AUC_0-t_ (µg·L^−1^·h)	79.6 ± 23.9	3.84 ±1.98
AUC_0-inf_ (µg·L^−1^·h)	82.6 ± 24.3	5.50 ± 2.93
Cl (L·kg·BW^−1^·h^−1^)	163 ± 52.2	3210 ± 2450
Vd (L·kg^−1^·BW)	1440 ± 417	22,400 ± 14,800

Tmax: time to reach maximum concentration of AFB_1_ in plasma. Cmax: maximum plasma concentration. T_1/2_Elim: terminal elimination half-life. AUC: area under the plasma concentration–time curve. Cl: total plasma clearance. Vd: volume of distribution.

**Table 3:** The total content of AFB_1_ and AFM_1_ in both feces and urine of male donkeys.

Parameters	Mass Value (µg)
AFB_1_ intake	12,420 ± 270
Absorption rate (%)	96.56 ± 1.45
AFB_1_ excretion via urine	210.86 ± 46.39
AFM_1_ excretion via urine	195.20 ± 66.62
Total AFB_1_ excretion through urine (%)	3.28 ± 0.92
AFB_1_ excretion via feces	409.30 ± 172.57
AFM_1_ excretion via feces	17.89 ± 8.40
Total AFB_1_ excretion through feces (%)	3.44 ± 1.45

Absorption rate (%) = (AFB_1_ intake—total AFB_1_ egestion through feces)/AFB_1_ intake × 100. AFB_1_ egestion through urine (%) = total AFB_1_ egestion through urine/AFB_1_ intake × 100. Total AFB_1_ egestion through urine = sum of AFB_1_ + AFM_1_ egestion via urine. AFB_1_ egestion through feces (%) = total AFB_1_ egestion through feces/AFB_1_ intake × 100. Total AFB_1_ egestion through feces = sum of AFB_1_ + AFM_1_ excretion via feces.

**Table 4:** Comparison of toxicokinetic parameters between donkeys and other animals.

Toxicokinetic Parameters	Donkey	Cow	Rhesus Monkey	Broiler Chickens
AFB_1_(µg·kg^−1^·BW)	100	40	50	2000
Cmax (µg·L^−1^)	15.6 ± 6.88	13.8 ± 0.9	-	9.8
Tmax (h)	1.38 ± 0.89	0.58 ± 0.17	4	0.25
T_1/2_Elim (h)	6.65 ± 2.84	15.52 ± 0.51	0.61	-
AUC_0-inf_ (µg·L^−1^·h)	82.6 ± 24.3	2.16 ± 0.36	-	1.6
Cl (L·kg·BW^−1^·h^−1^)	163 ± 52.2	-	1.25	-
Vd (L·kg^−1^·BW)	1440 ± 417	-	-	-

Tmax: time to reach maximum concentration of AFB_1_ in plasma. Cmax: maximum plasma concentration. T_1/2_Elim: terminal elimination half-life. AUC: area under the plasma concentration–time curve. Cl: total plasma clearance. Vd: volume of distribution.

**Table 5:** Analytical conditions for the LC-MS/MS analyses used to determine AFB_1_, AFM_1_, and ^13^C_17_-AFB_1_.

**Instrument**	**UPLC; Waters ACQUITY**		
Chromatographic column	BEH Shield RP18 column 100 mm × 2.1 mm i.d., dp 1.7 µm		
Gradient elution program	Time (min)	A: 0.1% FA and 0.1 mM AmAc in water (%)	B: MeOH containing 0.1% FA (%)
	0	95	5
	0.5	95	5
	1.5	50	50
	2.5	20	80
	3.5	20	80
	4	2	98
	5	2	--
	5.5	95	5
	6	95	5
Oven temperature	40 °C		
Flow velocity	0.3 mL·min^−1^		
Inoculability quantity	1 μL		
Ion source	ESI+		
Capillary voltage	0.6 kV		
Cone voltage	42 V		
MRM transitions	313.1 > 285.0 * and 313.1 > 241.2 for AFB_1_ (CE 22 and 36) 329.2 > 229.1 * and 329.2 > 259.0 for AFM_1_ (CE 26 and 24) 330.1 > 301.0 * and 330.1 > 255.1 for ^13^C_17_-AFB_1_ (CE 25 and 36)		
Source temperature	150 °C		
Desolvation temperature	500 °C		
Desolvation gas flow	900 L·h^−1^		
Cone gas	150 L·h^−1^		

* Quantification transition.

## Data Availability

The original contributions presented in this study are included in the article. Further inquiries can be directed to the corresponding authors.

## References

[1] Asao T., Büchi G., Abdel-Kader M.M., Chang S.B., Wick E.L., Wogan G.N., Asao T., Büchi G., Abdel-Kader M.M., Chang S.B. (1963). Aflatoxins B and G. J. Am. Chem. Soc..

[2] Asao T., Buechi G., Abdelkader M., Chang S., Wick E., Wogan G., Kader M.A., Agerwick E. (1965). The Structures of Aflatoxins B and G. J. Am. Chem. Soc..

[3] Gallo A., Moschini M., Masoero F. (2016). Aflatoxins absorption in the gastro-intestinal tract and in the vaginal mucosa in lactating dairy cows. Ital. J. Anim. Sci..

[4] Khodaei D., Javanmardi F., Khaneghah A.M. (2021). The global overview of the occurrence of mycotoxins in cereals: A three-year survey. Curr. Opin. Food Sci..

[5] Busby W.F., Wogan G.N. (1979). Food-borne mycotoxins and alimentary mycotoxicoses. Food-Borne Infections and Intoxications.

[6] Congcong L., Xiangdong L., Jiao W., Xiangbo J., Qiuliang X. (2022). Research progress in toxicological effects and mechanism of aflatoxin B1 toxin. PeerJ.

[7] Capriotti A.L., Caruso G., Cavaliere C., Foglia P., Samperi R., Laganà A. (2012). Multiclass mycotoxin analysis in food, environmental and biological matrices with chromatography/mass spectrometry. Mass Spectrom. Rev..

[8] Yiannikouris A., Jouany J.P. (2002). Mycotoxins in feeds and their fate in animals: A review. Anim. Res..

[9] Chen L., Wen T., Cao A., Wang J., Pan H., Zhao R. (2023). Bile Acids Promote Hepatic Biotransformation and Excretion of Aflatoxin B1 in Broiler Chickens. Toxins.

[10] Desjardins A. (2003). Mycotoxins: Risks in plant, animal and human systems. AAHE-ERIC/High. Educ. Res. Rep..

[11] Zhang K., Lian S., Shen X., Zhao X., Zhao W., Li C. (2022). Recombinant porcine beta defensin 2 alleviates inflammatory responses induced by Escherichia coli in IPEC-J2 cells. Int. J. Biol. Macromol..

[12] Vaziriyan M., Banaee M., Haghi B.N., Mohiseni M. (2018). Effects of dietary exposure to aflatoxins on some plasma biochemical indices of common carp (*Cyprinus carpio*). Iran. J. Fish. Sci..

[13] Battacone G., Nudda A., Cannas A., Borlino A.C., Bomboi G., Pulina G. (2003). Excretion of Aflatoxin M1 in Milk of Dairy Ewes Treated with Different Doses of Aflatoxin B1. J. Dairy Sci..

[14] Corcuera L.A., Vettorazzi A., Arbillaga L., González-Peñas E., López de Cerain A. (2012). An approach to the toxicity and toxicokinetics of aflatoxin B1 and ochratoxin A after simultaneous oral administration to fasted F344 rats. Food Chem. Toxicol..

[15] Wong Z.A., Hsieh D.P.H. (1980). The comparative metabolism and toxicokinetics of aflatoxin B1 in the monkey, rat, and mouse. Toxicol. Appl. Pharmacol..

[16] Cysewski S.J., Pier A.C., Baetz A.L., Cheville N.F. (1982). Experimental equine aflatoxicosis. Toxicol. Appl. Pharmacol..

[17] Laura G., Walter M., Tecla C., Claudia S., Stefania C., Luciano P. (2015). AFM1 in Milk: Physical, Biological, and Prophylactic Methods to Mitigate Contamination. Toxins.

[18] Iqbal S.Z., Jinap S., Pirouz A.A., Faizal A.R.A. (2015). Aflatoxin M1 in milk and dairy products, occurrence and recent challenges: A review. Trends Food Sci. Technol..

[19] Londoño V.A.G., Boasso A.C., Paula M.C.Z.d., Garcia L.P., Scussel V.M., Resnik S., Pacín A. (2013). Aflatoxin M1 survey on randomly collected milk powder commercialized in Argentina and Brazil. Food Control.

[20] Suriyasathaporn W., Nakprasert W. (2012). Seasonal patterns of aflatoxin Ml contamination in commercial pasteurised milk from different areas in Thailand. Food Addit. Contam..

[21] Ostry V., Malir F., Toman J., Grosse Y. (2017). Mycotoxins as human carcinogens—The IARC Monographs classification. Mycotoxin Res..

[22] Strosnider H., Azziz-Baumgartner E., Banziger M., Bhat R.V., Breiman R., Brune M.N., DeCock K., Dilley A., Groopman J., Hell K. (2006). Workgroup report: Public health strategies for reducing aflatoxin exposure in developing countries. Environ. Health Perspect..

[23] Kensler T.W., Roebuck B.D., Wogan G.N., Groopman J.D. (2011). Aflatoxin: A 50-Year Odyssey of Mechanistic and Translational Toxicology. Toxicol. Sci..

[24] Shuaib F.M., Jolly P.E., Ehiri J.E., Jiang Y., Ellis W.O., Stiles J.K., Yatich N.J., Funkhouser E., Person S.D., Wilson C. (2010). Association between Anemia and Aflatoxin B1 Biomarker Levels among Pregnant Women in Kumasi, Ghana. Am. J. Trop. Med. Hyg..

[25] Eaton D., Gallagher E.P. (1994). Mechanisms of Aflatoxin Carcinogenesis. Annu. Rev. Pharmacol..

[26] Zitomer N.C., Awuor A.O., Widdowson M.A., Daniel J.H., Sternberg M.R., Rybak M.E., Mbidde E.K. (2021). Human aflatoxin exposure in Uganda: Estimates from a subset of the 2011 Uganda AIDS indicator survey (UAIS). Food Addit. Contam. Part A.

[27] Keeton J.T., Eddy S. (2004). Chemical and physical characteristics of meat. Encyclopedia of Meat Sciences.

[28] Mohammed H.Z., Grace M.T., Ndivho N., Nthabiseng S.A., Letlhogonolo S., Monnye M. (2022). The Possibility of Including Donkey Meat and Milk in the Food Chain: A Southern African Scenario. Animals.

[29] Wenqiong C., Jing X., Honglei Q., Qiugang M., Mingxia Z., Mengmeng L., Yandong Z., Tianqi W., Jingrong G., Huanfen Y. (2022). Differential proteomic analysis to identify potential biomarkers associated with quality traits of Dezhou donkey meat using a data-independent acquisition (DIA) strategy. LWT.

[30] Chai W., Wang L., Li T., Wang T., Wang X., Yan M., Zhu M., Gao J., Wang C., Ma Q. (2024). Liquid Chromatography–Mass Spectrometry-Based Metabolomics Reveals Dynamic Metabolite Changes during Early Postmortem Aging of Donkey Meat. Foods.

[31] Meena S., Meena G.S., Gautam P.B., Rai D.C., Kumari S. (2024). A comprehensive review on donkey milk and its products: Composition, functionality and processing aspects. Food Chem. Adv..

[32] Kang R., Qu H., Guo Y., Ji C., Cheng J., Wang Y., Huang S., Zhao L., Ji C., Ma Q. (2023). Toxicokinetics of Deoxynivalenol in Dezhou Male Donkeys after Oral Administration. Toxins.

[33] Kang R., Qu H., Guo Y., Zhang M., Fu T., Huang S., Zhao L., Zhang J., Ji C., Ma Q. (2023). Toxicokinetics of a Single Oral Dose of OTA on Dezhou Male Donkeys. Toxins.

[34] Qu H., Zheng Y., Kang R., Feng Y., Li P., Wang Y., Cheng J., Ji C., Chai W., Ma Q. (2024). Toxicokinetics of Zearalenone following Oral Administration in Female Dezhou Donkeys. Toxins.

[35] Wang Y.C., Chiang J.H., Hsu H.C., Tsai C.H. (2019). Decreased fracture incidence with traditional Chinese medicine therapy in patients with osteoporosis: A nationwide population-based cohort study. BMC Complement. Altern. Med..

[36] Xue L., Feng S., Liping G., Baojian H., Daoyuan L., Lianli C. (2017). Species-specific identification of collagen components in Colla corii asini using a nano-liquid chromatography tandem mass spectrometry proteomics approach. Int. J. Nanomed..

[37] Jubert C., Mata J., Bench G., Dashwood R., Pereira C., Tracewell W., Turteltaub K., Williams D., Bailey G. (2009). Effects of Chlorophyll and Chlorophyllin on Low-Dose Aflatoxin B1 Pharmacokinetics in Human Volunteers. Cancer Prev. Res..

[38] Sun P., Lootens O., Kabeta T., Reckelbus D., Furman N., Cao X., Zhang S., Antonissen G., Croubels S., De Boevre M. (2024). Exploration of Cytochrome P450-Related Interactions between Aflatoxin B1 and Tiamulin in Broiler Chickens. Toxins.

[39] Masoero F., Gallo A., Moschini M., Piva G., Diaz D. (2007). Carryover of aflatoxin from feed to milk in dairy cows with low or high somatic cell counts. Anim. Int. J. Anim. Biosci..

[40] Bastaki S.A., Osman N., Kochiyil J., Shafiullah M., Padmanabhan R., Abdulrazzaq Y.M. (2010). Toxicokinetics of Aflatoxin in Pregnant Mice. Int. J. Toxicol..

[41] Saunders E. (2006). A textbook of the diseases of cattle, horses, sheep, pigs and goats. Veterinary Medicine.

[42] Applebaum R.S., Brackett R.E., Wiseman D.W., Marth E.H. (1982). Responses of Dairy Cows to Dietary Aflatoxin: Feed Intake and Yield, Toxin Content, and Quality of Milk of Cows Treated with Pure and Impure Aflatoxin. J. Dairy Sci..

[43] Gross-Steinmeyer K., Eaton D.L. (2012). Dietary modulation of the biotransformation and genotoxicity of aflatoxin B1. Toxicology.

[44] Lakritz J., Winder B.S., Noorouz-Zadeh J., Huang T.L., Buckpitt A.R., Hammock B.D., Plopper C.G. (2000). Hepatic and pulmonary enzyme activities in horses. Am. J. Vet. Res..

[45] Wild C., Garner R., Montesano R., Tursi F. (1986). Aflatoxin B1 binding to plasma albumin and liver DNA upon chronic administration to rats. Carcinogenesis.

[46] Jager A., Tonin F., Souto P., Privatti R., Oliveira C. (2011). Determination of Urinary Biomarkers for Assessment of Short-Term Human Exposure to Aflatoxins in So Paulo, Brazil. Toxins.

[47] Mccoy L.F., Scholl P.F., Schleicher R.L., Groopman J.D., Powers C.D., Pfeiffer C.M. (2010). Analysis of aflatoxin B1-lysine adduct in serum using isotope-dilution liquid chromatography/tandem mass spectrometry. Rapid Commun. Mass Spectrom..

[48] Rustemeyer S.M., Lamberson W.R., Ledoux D.R., Rottinghaus G.E., Cammack K.M. (2010). Effects of dietary aflatoxin on the health and performance of growing barrows. J. Anim. Sci..

[49] Gratz S., Taubel M., Juvonen R.O., Viluksela M., Turner P.C., Mykkanen H., El-Nezami H. (2006). Lactobacillus rhamnosus Strain GG Modulates Intestinal Absorption, Fecal Excretion, and Toxicity of Aflatoxin B1 in Rats. Appl. Environ. Microbiol..

[50] Fernández A., Belío R., Ramos J.J., Sanz M.C., Sáez T. (1997). Aflatoxins and their Metabolites in the Tissues, Faeces and Urine from Lambs Feeding on an Aflatoxin-Contaminated Diet. J. Sci. Food Agric..

[51] Firmin S., Morgavi D.P., Yiannikouris A., Boudra H. (2011). Effectiveness of modified yeast cell wall extracts to reduce aflatoxin B1 absorption in dairy ewes. J. Dairy Sci..

[52] Caloni F., Cortinovis C. (2011). Toxicological effects of aflatoxins in horses. Vet. J..

[53] Wang R., Su X., Wang P., Zhang W., Xue Y., Li Y. (2017). Simultaneous detection of 21 kinds of mycotoxins and their metabolites in animal plasma with impurity adsorption purification followed by liquid chromatography-tandem mass spectrometry. Chin. J. Anal. Chem..

